# Dr. James Hemstreet

**Published:** 1914-09

**Authors:** 


Dr. James Hemstreet (not listed in State Directory) died at Poland, Herkimer Co., N. Y., July 21, aged 90.

